# Case of Retroverted Uterus

**Published:** 1846-01

**Authors:** U. P. Golliday

**Affiliations:** Kickapootown, Ill.


*Case of Retroverted Uterus.* By U. P. Golliday, of Kickapootown, Ill.

On the evening of Friday, 15th of August, last, I was requested to see Mrs. A. J———, who, as the messenger said, "was in severe pain, and then would be easy for a while." I found her apparently in strong labor, and was informed by a lady present, (who by the way, was an accoucheur of some eminence among her neighbors) that there was something very strange and peculiar in the case. Examination per vaginam revealed a tumor low in the Pelvic excavation, the posterior wall of the vagina could be traced from the fourchette to a point above the pubes, the tumor pressing with a moderate force upon the postero-superior surface of the pubes, but not sufficiently to prevent examination, and yet the os uteri could not be found. Examination by the rectum showed the tumor to be moveable, it could be quite easily tilted upon the end of the finger, except during a pain, when it seemed to sink lower in the pelvis. Neither urine nor fæces had been passed for some time. The patient was placed upon the face with the knees drawn up under the abdomen. The tumor could now be easily moved, but the portion resting upon the rectum, could not, by any reasonable force, be moved above the promontory of the sacrum. The position was changed, and urine and fæces passed without difficulty. Those circumstances led me to believe the case one of retroverted uterus. It was evident there was not an impacted condition of the organ; *the seventh month had passed*, and from the necessary distension of the uterus, it appeared evident to me, that no impaction could take place, nor could the position of the uterus be changed so as to bring the os uteri into the proper place, without an evacuation of its fluid contents. I thought of puncturing the uterus as the only certain means of relieving the patient; but as her strength still seemed firm, I considered it best to continue the means before used, (pressure upwards upon the fundus through the rectum and traction upon the cervix through the vagina,) with such collateral aid as circumstances would admit of. After unavailing efforts had been used for some time, an opiate was administered, and she was left for the night. As she appeared comparatively comfortable in the morning, I left her with such directions as were judged necessary for the day, and returned home. Recalled in the evening, my friend Dr. H. S. Hubbard, of Toulon, went with me. He fully concurred with the opinion previously formed of the case, and every effort was made to relieve the patient, that we considered prudent, but without benefit. Our patient was evidently getting worse, and the propriety of puncturing the uterus through the posterior wall of the vagina, was seriously discussed; but not wishing to have all the responsibility of such a case resting upon ourselves, whilst additional counsel could be easily procured, we sent for J. C. Frye, M.D., of the city of Peoria, determining, in the mean time, to use all efforts that a prudent regard for the safety of our patient would justify.

We had observed that when placed upon the face, our patient was easier, the pains did not appear to force the uterus as low in the pelvis as when she was placed on her back or inclined to one side. We had also learned that she had been in a similar situation twice before, and that spontaneous rupture of the membranes, and speedy delivery of the fœtus had taken place. Impressed with these facts, we directed the patient to be placed upon the back, that the uterine effort being stronger in that position, might either forcibly rupture the membranes, and bring about a proper position of the womb for delivery; or reduce the tumor so low in the pelvis, that if puncture were determined on, the parts might be in a proper position for it.

It was but a short time until spontaneous rupture of the membranes occurred, and the woman was delivered of a dead child. She recovered as soon as could have been expected, under the most favorable circumstances.

[Dr. G. argues at considerable length the propriety of the proposed puncture of the womb, and quotes many cases of rupture and extirpation of the organ in which the patients recovered, to prove that it would not be so dangerous as some have supposed. But, as Dr. Ashwell, in his late work on the diseases peculiar to women, speaks at length on the propriety of tapping the uterus through the vagina or rectum in extreme cases, we think better to refer the reader to this excellent work. Blundell recommends the operation "when the catheter could not be introduced, nor the rectum emptied," and suggests that a very small trochar and canula should be used, so that the wound would resemble acupuncturation.

The operation was first suggested by Dr. William Hunter, and has been twice performed,—once on the continent, and once by Mr. Baynham of Birmingham, whose case was reported in the Edin. Med. and Surg. Jour. in 1830. It proved entirely successful under adverse circumstances.

As Dr. G's patient had been in the same condition twice before, it would be an interesting enquiry to ascertain what was the cause that determined the mal-position; was it a peculiarity in *conformation?* or were the causes accidental, occurring thrice in succession? J. E.]

